# Review of Climate, Landscape, and Viral Genetics as Drivers of the Japanese Encephalitis Virus Ecology

**DOI:** 10.1371/journal.pntd.0002208

**Published:** 2013-09-12

**Authors:** Guillaume Le Flohic, Vincent Porphyre, Philippe Barbazan, Jean-Paul Gonzalez

**Affiliations:** 1 UR Ecologie et Santé, Centre International de Recherches Médicales de Franceville, CIRMF, BP 769, Franceville, Gabon; 2 Centre de Coopération Internationale en Recherche Agronomique pour le Développement, CIRAD, Montpellier, France; 3 Institut de Recherche pour le Développement, IRD, Marseille, France; 4 Ministère des Affaires Etrangères et Européennes, Mission de Coopération, Libreville, Gabon; 5 Metabiota, Washington, District of Columbia, United States of America; Centers for Disease Control and Prevention, United States of America

## Abstract

The Japanese encephalitis virus (JEV), an arthropod-born *Flavivirus*, is the major cause of viral encephalitis, responsible for 10,000–15,000 deaths each year, yet is a neglected tropical disease. Since the JEV distribution area has been large and continuously extending toward new Asian and Australasian regions, it is considered an emerging and reemerging pathogen. Despite large effective immunization campaigns, Japanese encephalitis remains a disease of global health concern. JEV zoonotic transmission cycles may be either wild or domestic: the first involves wading birds as wild amplifying hosts; the second involves pigs as the main domestic amplifying hosts. *Culex* mosquito species, especially *Cx. tritaeniorhynchus*, are the main competent vectors. Although five JEV genotypes circulate, neither clear-cut genotype-phenotype relationship nor clear variations in genotype fitness to hosts or vectors have been identified. Instead, the molecular epidemiology appears highly dependent on vectors, hosts' biology, and on a set of environmental factors. At global scale, climate, land cover, and land use, otherwise strongly dependent on human activities, affect the abundance of JEV vectors, and of wild and domestic hosts. Chiefly, the increase of rice-cultivated surface, intensively used by wading birds, and of pig production in Asia has provided a high availability of resources to mosquito vectors, enhancing the JEV maintenance, amplification, and transmission. At fine scale, the characteristics (density, size, spatial arrangement) of three landscape elements (paddy fields, pig farms, human habitations) facilitate or impede movement of vectors, then determine how the JEV interacts with hosts and vectors and ultimately the infection risk to humans. If the JEV is introduced in a favorable landscape, either by live infected animals or by vectors, then the virus can emerge and become a major threat for human health. Multidisciplinary research is essential to shed light on the biological mechanisms involved in the emergence, spread, reemergence, and genotypic changes of JEV.

## Introduction

The incidence of zoonotic diseases, transmitted to humans from wild or domestic animals, has noticeably increased during the past few decades and currently represents 70% or more of emerging diseases [1]. Japanese encephalitis virus (JEV), an arbovirus of the *Flavivirus* genus, family Flaviviridae, is transmitted by mosquitoes from animals to humans. In humans, this zoonotic disease is the largest worldwide cause of epidemic viral encephalitis [2], [3]. The pathogenesis of the JEV and the clinical manifestations of the disease, including severe neurological syndromes, depend on several factors that have been deeply reviewed elsewhere [4], [5]. The incubation period ranges from 5 to 15 days; JE infections are lethal in about 25–30% of cases, mostly in infants, and lead to permanent sequelae in about 50% of cases.

JE was first described in 1871 in Japan, and first characterized in 1935 [6]. Despite an effective vaccine developed in 1941, and the subsequent national immunization campaigns that have greatly reduced the incidence of JE in several countries, sporadic cases continue to be reported, and JEV continues to spread widely in South, East, and Southeast Asia and Australasia [7]. Currently, more than three billion people live in JE-endemic countries [5]. There, though people of all ages may be exposed to JEV, the JE incidence is higher in children because most adults are immune [8]. Now, about 68,000 JE cases are estimated to occur annually, causing at least 10,000–15,000 deaths in more than 20 Australasian countries [8], [9].

## Aims and Methodology

This review aims at offering an overview of the factors affecting JEV epidemiology. It discusses the current state of knowledge on JEV diversity, molecular epidemiology, and ecology, and examines how environmental variables and modifications, especially those associated with agriculture, affect the landscape characteristics and JEV ecology. The literature review was achieved by using both general web search engines and scientific web search engines such as PubMed, Springerlink, ScienceDirect, and Web of Science. It focused on the literature available on virology, molecular and spatial epidemiology, entomology, and landscape and behavioral ecology.

## JEV Genotypes

Five JEV genotypes have been distinguished by nucleotide sequencing of the *C/PrM* and *E* (capsid, precursor membrane, and envelope) genes [10]. Genotypes I, II, and III are distributed throughout Asia, while genotype IV is restricted to Eastern Indonesia [11]. Genotype V was long restricted to Malaysia but isolates were recently found in China and in the Republic of Korea [12], [13]. Genotypes IV and V form the oldest JEV lineage, which appears to have originated from an ancestral virus in the Indonesian-Malaysian region. JEV therefore probably originally spread from this region [4], [11]. Genotypes I, II, and III are the most prevalent, having accounted for 98% of the strains isolated from 1935 to 2009 [14].

## JEV Molecular Epidemiology

Until recently, genotypes I and III were mostly associated with epidemic diseases in temperate regions of Asia, while genotypes II and IV were associated with endemic diseases in tropical regions. It was therefore postulated that the JEV epidemiology differed with genotypes [15]. However, more recent observations show that genotypes can be found indifferently within epidemic or endemic areas. Indeed, genotype I has been isolated in Australasia within the tropical region [16], genotype II has been shown to have circulated in the Republic of Korea within the temperate region before the 1970s [17], [18], and genotype III, the only one in India so far, has circulated both in the south Indian peninsula (characterized by endemic activity) and in the north (characterized by epidemic activity) [7], [19].

**Figure 1:**
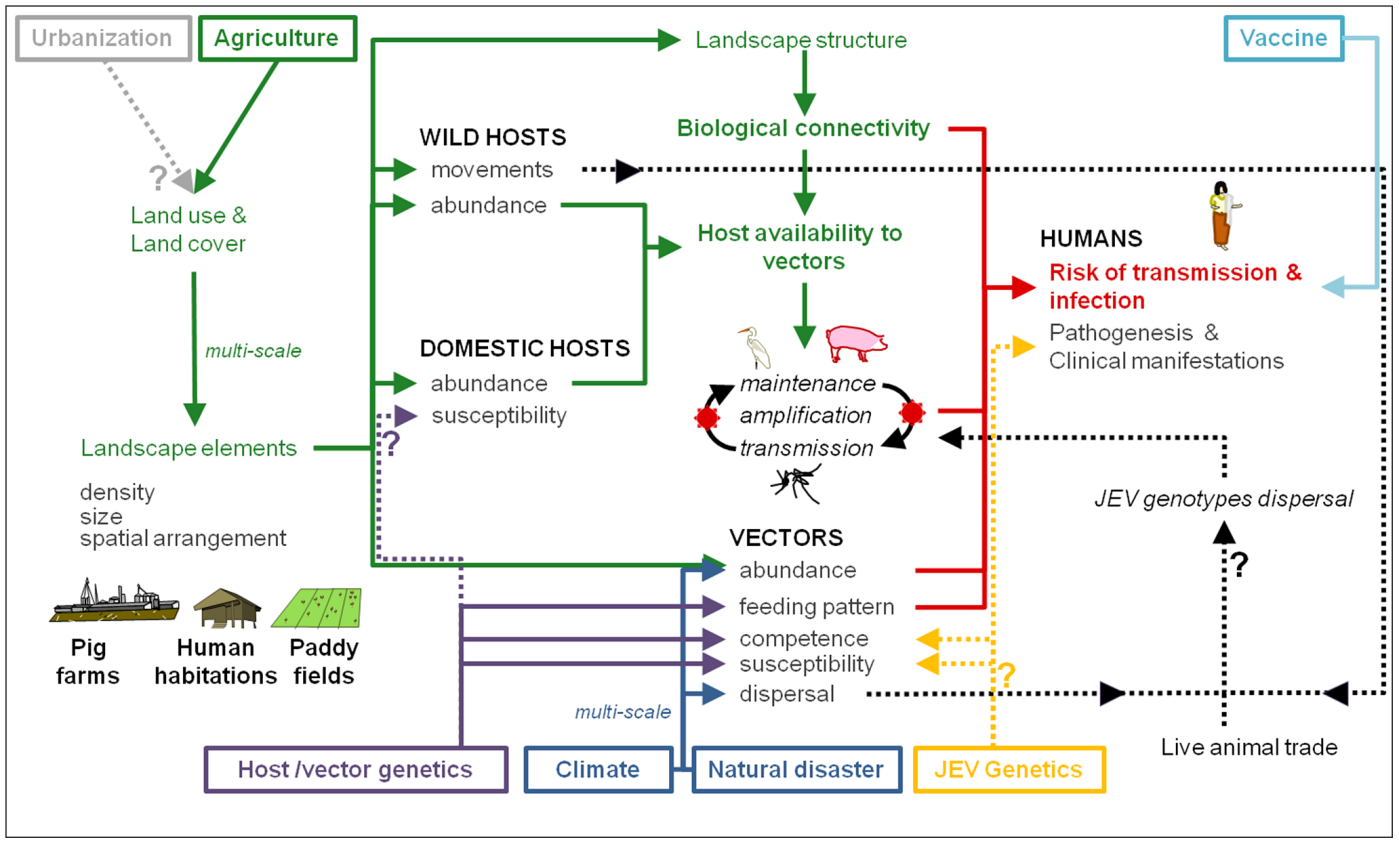
Overview of the drivers of the JEV ecology and epidemiology. Arrows from one driver (ex: climate) to another (ex: vectors abundance) illustrate the influence of the first on the second. Lines are dotted when the link between two drivers is uncertain. Question tags underscore uncertain aspects of the JEV cycle. The JEV is represented in red in the zoonotic cycle in the middle of the figure. Humans, as they stay outside of the JEV cycle, do not enter the cycle. Major drivers influencing the JEV ecology and ultimately the epidemiology in humans are framed. These drivers and their influences are colored as follow: in green, agriculture; in blue, climate and natural disasters; in purple, host/vector genetics; in yellow, JEV genetics; urbanization, which is a priori not relevant to the JEV life cycle, appears in gray. Vaccine, as one major nonenvironmental driver influencing the risk of JEV transmission and infection to humans, is in light blue. “multi-scale” is annotated when one driver has a multiple spatial scales effect on another.

Differences in the JEV genome might explain phenotypic variations of the disease. Experimental changes in the *E* gene have indeed been shown to be associated with loss or gain of neuroinvasiveness and neurovirulence in mice and monkeys [20]–[23]. Even so, experimental studies of the neurovirulence of wild JEV strains in mice showed no clear-cut genotype-phenotype relationship [24], and there is still no firm evidence that the JEV genotypes circulating differ in their virulence (Figure 1). However, the dengue virus type 2, another closely related *Flavivirus*, has been shown to possess interesting genotype-dependent characteristics such as human viral pathogenicity or midgut viral replication in *Aedes aegypti* [25], [26]. The JEV molecular markers previously and currently studied might not be sufficient and need further investigation.

Rather than by viral genetic determinants solely, the JEV epidemiological pattern appears to be actually influenced by a complex set of variables, including several environmental, ecological, and immunological factors [7] (Figure 1).

## JEV Spatial Epidemiology

The distribution area of JEV has consistently enlarged. Sometimes, the genotypes of JEV isolates changed locally. In northern Vietnam and Thailand, a shift from genotype II to genotype I was reported [27], [28]. In northern temperate regions (i.e., Japan, South Korea, and Northeast China), genotype I progressively replaced genotype III and became the main genotype [29]. It is now very probably the most widespread genotype in Asia, which was otherwise found to be lethal for humans [9], [29]–[33]. However, to date, vaccines are only derived from genotype III strains; whereas protective levels of cross-reactive neutralizing antibodies of these vaccines were found against the various circulating genotypes, variations between genotypes call for further studies [34], [35]. In particular, since the immune response against genotype I was less pronounced, its duration should be addressed [34].

## JEV Ecology: Vector- and Host-Virus Interactions

### Vectors

Over 30 mosquito species (family Culicidae) belonging to the *Aedes*, *Anopheles*, *Armigeres*, *Culex*, and *Mansonia* genera are recognized to potentially carry JEV, but not all are equally competent for virus transmission [7]. Susceptibility and competence vary among and within mosquito species [36], but also possibly covariate with genotypes, as has been shown for dengue [26], [37] and Chikungunya virus strains [38]. However, no variations in vector competence and transmissibility by various mosquito species for different JEV genotypes have yet been demonstrated.

*Culex* species are the most competent JEV vectors, in the same way that *Aedes* species are the most competent Dengue viruses vectors [39]. This low plasticity of the virus to vectors may be explained by the vector ecology, especially host-feeding preferences, as few species feed on JEV reservoir-amplifying hosts. For example, in mainland Australia *Cx annulirostris*, a competent JEV vector, feeds predominantly on marsupials, and thus remains outside the JEV transmission cycle [40], [41]. JEV is transmitted by *Cx. annulirostris*, *Cx. annulus*, *Cx. fuscocephala*, *Cx. gelidus*, *Cx. sitiens*, *Cx. vishnui* species complex, and the rice field–breeding mosquitoes *Culex tritaeniorhynchus* [42]. *Cx. tritaeniorhynchus* is the major JEV vector because it is highly competent and is largely distributed all over the JEV-endemic regions [3], [43]. JEV vectors are highly zoophilic, tending to prefer wading birds, cattle, and pigs to humans [42], [44], [45]. Therefore, vector trophic preferences are a major ecological factor affecting the fitness of the virus.

Variations of competence may be due to differences in the susceptibility to infection of mosquito midgut and salivary glands, and then to differences in the efficiency of virus secretion and transmission [46]. Susceptibility to JEV infection is partly dependent on the *E* gene sequence of the virus. This gene encodes a protein involved first in virus cellular entry through attachment to membrane receptors and second in the fusion of both JEV and host-cell membranes [47]. However, despite some study on the subject [48], no data demonstrates there are important variations in vector competence and transmissibility between various mosquito species for different JEV genotypes.

Hosts also play a central role in virus ecology, according to their viraemia and availability within the immediate vector flight range. Ultimately, two types of JEV transmission cycle can be drawn: a bird-associated wild cycle and a pig-associated rural domestic cycle.

### Bird-Associated Wild Cycle

Although in the wild a wide range of animals (bats, birds, rodents, snakes, wild boars…) may be infected by JEV, with high seroprevalence rates in several mammal and bird species, most of them are dead-end hosts and are unable to infect mosquitoes [2], [3]. However, over 90 bird species are known to be amplifying and reservoir hosts of JEV. Among them, wild wading birds, in particular egrets (*Egretta garzetta*) and herons (*Nycticorax nycticorax*) of the Ardeidae family, are highly susceptible to JEV infection. They represent the primary effective animal hosts, since they have a high virus titer, and are an outstanding source of infection for mosquitoes [3], [7], [9]. As many of them are widely distributed and migratory, ardeid species are suspected of being responsible for several virus introductions, from China to Japan, and from the Indochinese peninsula to northern Asia [27]. Several additional wild animal species are considered part of alternative JEV cycles: wild boars have been suspected to be the source of sporadic human cases of JE in Singapore [49], wild peridomestic mammals (raccoons, wild boars, raccoon dogs) in Japan, and flying foxes in Australia tested positive for JEV reacting antibodies [50], [51]. In China, JEV has been isolated from two bat species [52].

In the wild cycle, JEV vectors, although highly zoophilic, are largely opportunistic blood feeders and their feeding pattern relies more likely on the host availability within the given landscape than on innate preferences [40], [53]. Humans in the vicinity of this cycle could be accidentally infected.

### Pig-Associated Rural Domestic Cycle

In the JEV rural domestic cycle, cattle, chicken, dogs, ducks, goats, horses, and pigs may be infected, but only a few of them are efficient virus amplifiers [2]. This domestic cycle occurs mainly within rural landscapes, with pigs acting as the primary domestic amplifying host. Some other domestic mammals (cattle, dogs, goats) also display high seroprevalence rates, as pigs do; however, pigs replicate the virus quickly and have the highest viraemia [3], [54]. Due to intensive pig farming in East and Southeast Asia (http://faostat3.fao.org/), pigs are the main component of the domestic cycle [6]. In particular, the industrialization of pig farming, which has broadly developed for several decades in many countries (China, Myanmar, Thailand, Vietnam) [6], has enhanced the amplification of the JEV within dense pig herds, and thus contributed to increase the risk of JEV transmission. Therefore, the high proximity between humans and livestock became the main risk factor for human infection [6], [55].

Interaction between domestic host and pathogen depends on several host genetic, physiologic, and agricultural factors. First, for many diseases, resistance (defined as the ability to resist infection or moderate pathogen lifecycle) and tolerance (defined as an asymptomatic infection) in livestock have a genetic component, including breeds' genetic traits ([56], [57]; examples given by Gibson [58]). While resistance to infection limits the circulation and maintenance of pathogens in a population, tolerance on the contrary does not limit the virus transmission [56]. The replacement of indigenous breeds by exotic ones (Landrace, Large White, or Duroc) might potentially have changed the susceptibility to JEV infection in domestic pigs. However, data on susceptibility or differences in the ability to amplify JEV between exotic imported or indigenous pig breeds are lacking. Second, variations in the attractiveness of pig hosts between farms depends on physiological and agricultural factors (e.g., sows are more often bitten than piglets, as are encaged pigs) [59], [60]. Third, the intensity of circulation in farms depends on the amount of pigs reared by farm, their age at slaughter, their reproduction rate, and the vaccination effort [59]. Subsequently, the epidemiology of JEV in the domestic reservoir host follows a complex multifactorial pattern that needs to be brought to light.

The mosquito vector species present in the wild transmission cycle are also greatly implicated in the rural domestic cycle. Although most mosquito species usually consume bird, cattle, and pig blood [61], *Cx. quinquefasciatus*, whereas opportunistic, seems also very anthropophilic on account of its use of various domestic water collections and wastewater from pig farms as breeding sites [2], [45], [55], [62], [63]. Because it has been shown to naturally carry the JEV and to be experimentally capable of transmitting it [48], [63], *Cx. quinquefasciatus* is another potential vector of the JEV domestic cycle. It represents an important potential source of infection for humans, so much the more it has emerged in areas where it was absent before following urbanization and human expansion, and increases in number in urban households with human density and pig keeping [64], [65].

### Evolutionary Ecology of the JEV

Until recently, the factors influencing the historical and geographical emergence and spread of JEV genotypes were not clearly understood. In fact, genotypes' emergence and spread appear to have a complex pattern relevant with the role of environmental factors. Some emerge in areas where they were absent before (e.g., genotype V in Tibet, a cold and high-altitude region that was considered free of JEV [12], [66], and genotype I in the Australasian region [16]), whereas some replace the dominant genotype (e.g., genotype III to genotype I shift).

It is thought that these changes are due to the JEV fitness to a new competent vector (e.g., genotype V, besides being isolated from *Cx. tritaeniorhynchus*, seems associated with a new JEV vector in the Republic of Korea: *Cx. bitaeniorhynchus* [13]) or to new host availability (e.g., increasing pig farming in Tibet [12]). More likely, environmental factors (e.g., mosquito dispersal by wind in Australia), animal ecology (e.g., migration of animals), human activity (e.g., pig trade introducing genotype I in Australia [16]), and climatic changes [67] are variables independent to JEV genetics that may modify the distribution area of JEV and its genotypes.

## Environmental Factors: Drivers of JEV Epidemiology

The (re)emergence of vector-borne and zoonotic diseases as well as variations in disease risk and incidence are strongly driven by environmental factors. Among the most relevant factors are climate but also land cover and land use, respectively the biophysical attributes of the earth's surface and the human purpose or intent applied to these attributes [68]. However, these variables occur at several different geographic ranges, and hence multi-scale studies are a major stake for the understanding of the spatial epidemiology of diseases [69].

For a long time, JEV epidemiology has exclusively been broached at the global scale and primarily on the basis of climatic variables (i.e., rainfall and temperature) because they indeed strongly affect vector density [67]. Recently, Miller et al. [70] identified an optimal range of temperature during the wet season that is favorable to the *Cx. tritaeniorhyncus* biology. As they also found that most of JE cases were located in areas of high probability of vectors, they underlined the link between climatic covariables, vector ecology, and human health. Moreover, temperature might affect the competence of a vector-genotype couple over another, as shown for the West Nile virus [71].

In our previous study [14], six Asian and Australasian regions were identified according to anthropogenic, biological, geographic, and physical factors including not only climatic conditions, but also biomes and land use. The regional history of JEV genotypes was analyzed (i.e., the number of JEV isolates and the proportion of each genotype). Then we emphasized the two regions of intensive JEV circulation: the Paleartic biogeographic realm [72] comprising Eastern Russia, Japan, Korea, and Northern China, and a tropical region characterized by large cover of rice fields comprising the Indochinese peninsula (Cambodia, Lao PDR, Myanmar, Thailand, and Vietnam), South China, and Taiwan [73].

Actually, land cover and land use changes have been driving the JE risk and incidence. In Asia, paddy field surfaces have constantly extended since the early 1960s (http://faostat3.fao.org/). Given paddy fields provide long-term *Culex* sp. breeding sites and attract many wading birds for foraging and resting, they enhance the circulation and expansion of mosquito and wading bird populations [74]. Thus, in areas where rice-irrigated farming is widespread, the JEV transmission might become less dependent on rainfall [75], [76]. Likewise, because the amount of live pig heads has increased since the 1960s (http://faostat3.fao.org/), both backyard and industrial pig farming have provided a continuous increasing, outstanding potential source of blood meals for mosquitoes.

Additionally, a finer-scale spatial analysis (i.e., local or regional landscape) helps to better picture how landscape structure affect the JEV epidemiology. At fine scale, JEV ecology is indeed largely affected by both land cover and land use as these factors affect vectors' and hosts' ecology (Figure 1). Once the JEV is introduced, its ecological sustainability indeed relies on its growth within the susceptible host population and on its transmission between hosts through vectors. Then the risk of incidental infection to humans depends on the likelihood of disease transmission within the surrounding landscape [77]. To be transmitted to humans, JEV needs: 1) competent mosquito vectors, 2) reservoir-amplifying hosts, and 3) nonimmune humans within the range of the JEV-competent vector and animal hosts. In this process, the three major landscape elements are paddy fields, pig farms, and human habitations. In the landscape, they are distributed in habitat patches. Their density, size, and spatial arrangement are key factors determining the degree to which the landscape facilitates or impedes movement of vectors among them (i.e., the landscape connectivity) [78] (Figure 1). Within a given landscape, this biological connectivity may strongly affect the JEV transmission dynamics.

## Nonenvironmental Factors of JEV Epidemiology

Vaccination is the most effective way to reduce the incidence of JE in humans, yet it has no effect on the JEV transmission cycle. Vaccination of livestock, especially pigs, would in contrast reduce the amplification of the virus, the rate of mosquito infection, and subsequently the risk of transmission to humans. Yet, vaccination of pigs is generally not used to prevent JE because it is costly, hardly feasible logistically, and not necessarily effective in piglets (they must be immunized after the disappearance of maternal antibodies) [6], [79]. Moreover, pigs represent a relevant sentinel model, the surveillance of which could predict a potential JE outbreak in a human population nearby [54]. Immunizing sentinel pigs would also impede the detection of such a threat. Other factors have strong effects on the JEV and genotype circulation within infected landscapes and their emergence in nonendemic landscapes and regions. There are, among them, the movements of either naive or infected animals such as the migrations of flying vertebrates and trading and transportation of domestic animals. These movements modify human exposure to JEV and thus require the particular attention of the health system. In southeastern Asia, trade of live animals occurs between farms, local markets, and more importantly within the Indochinese peninsula and China, and also to Hong Kong and Singapore [80]. Viremic birds, for instance, may have been responsible for JEV spread and introductions [4], [27] into India [81], Taiwan [82], or Papua New Guinea [83]. Over long distances, migratory birds are the most likely spreader of the JEV as some species, such as *Egretta garzetta* and *Nycticorax nycticorax*, have a complex migration system over a large geographical area (http://maps.iucnredlist.org). However, the large-scale movement patterns of the main wading birds species implicated in JEV transmission are little known. Additionally, JEV may disperse through wind-blown infected mosquitoes, as is assumed to be the cause of the JEV introduction from Papua New Guinea to north Australia [84] or from China to Japan [85].

Moreover, there is no evidence that JEV requires adaptation to shift between the bird-associated wild cycle and the pig-associated rural domestic cycle [2], [3]. The JEV may easily be transferred from one to another if competent vectors feeding on both wild and domestic hosts are present in JEV-infected areas where both hosts are in close proximity.

## Conclusion

For more than a half century, human activities have led to changes of land use and land cover and subsequent deep environmental changes of habitats. These modifications have modified the risk of infection for humans. The ecology of arthropod-borne diseases, noticeably JE, remains complex since they are highly dependent on various biotic and abiotic environmental factors and on the spatial scale of study. To assess and predict JE emergence therefore remains difficult. Multidisciplinary research focusing on virology, molecular and spatial epidemiology, landscape and behavioral ecology, history, and socioeconomic studies are essential to shed light on the biological mechanisms involved in the emergence, spread, reemergence, and genotypic changes of JEV.

**Five Key Articles in the Field**

1. van den Hurk AF, Ritchie SA, Mackenzie JS (2009) Ecology and geographical expansion of Japanese encephalitis virus. Annu Rev Entomol 54: 17–35.
2. Weaver SC, Barrett ADT (2004) Transmission cycles, host range, evolution and emergence of arboviral disease. Nat Rev Microbiol 2: 789–801.
3. Mackenzie JS, Gubler DJ, Petersen LR (2004) Emerging flaviviruses: the spread and resurgence of Japanese encephalitis, West Nile and dengue viruses. Nat Med 10: S98–109.
4. Solomon T (2006) Control of Japanese encephalitis — within our grasp? N Engl J Med 355: 869–871.
5. Solomon T, Ni H, Beasley DWC, Ekkelenkamp M, Cardosa MJ, et al. (2003) Origin and evolution of Japanese encephalitis virus in Southeast Asia. J Virol 77: 3091–3098.

**Key Learning Points**

1. The Japanese encephalitis virus epidemiology is strongly dependant on the ecology of both amplifying hosts and mosquito vectors, which themselves are affected not only by natural environmental factors but also by human activities.
2. The spatial epidemiology of the JEV needs to be investigated at global and landscape scales, as human-induced land use and land cover have effects on the species distribution area, population density, species community structure, and interactions between vectors, hosts, and humans.
3. Though the fitness of the virus to hosts and vectors appears little dependent on genetic factors (genotypes), knowledge of other closely related arboviruses suggests that the epidemiological implications of genotypes might be underestimated.
4. The introduction, spread, emergence, and persistence of JEV zoonotic cycles in a new region may be predicted according to the characteristics of the landscape at various spatial scales.
